# Involvement of basolateral amygdala‐rostral anterior cingulate cortex in mechanical allodynia and anxiety‐like behaviors and potential mechanisms of electroacupuncture

**DOI:** 10.1111/cns.70035

**Published:** 2024-09-15

**Authors:** Yuerong Chen, Siyuan Tong, Yingling Xu, Yunyun Xu, Zonglin Wu, Xixiao Zhu, Xirui Wang, Chaoran Li, Chalian Lin, Xiaoyu Li, Chi Zhang, Yifang Wang, Xiaomei Shao, Jianqiao Fang, Yuanyuan Wu

**Affiliations:** ^1^ Key Laboratory of Acupuncture and Neurology of Zhejiang Province, Department of Neurobiology and Acupuncture Research The Third Clinical Medical College, Zhejiang Chinese Medical University Hangzhou China; ^2^ NHC and CAMS Key Laboratory of Medical Neurobiology, MOE Frontier Science Center for Brain Research and Brain‐Machine Integration, School of Brain Science and Brain Medicine Zhejiang University Hangzhou China; ^3^ Liangzhu Laboratory Zhejiang University Medical Center Hangzhou China; ^4^ Tuina Department Hangzhou Red Cross Hospital Hangzhou China

**Keywords:** anxiety, basolateral amygdala, chronic pain, electroacupuncture, rostral anterior cingulate cortex

## Abstract

**Aims:**

Chronic pain is highly associated with anxiety. Electroacupuncture (EA) is effective in relieving pain and anxiety. Currently, little is known about the neural mechanisms underlying the comorbidity of chronic pain and anxiety and the EA mechanism. This study investigated a potential neural circuit underlying the comorbid and EA mechanisms.

**Methods:**

Spared nerve injury (SNI) surgery established the chronic neuropathic pain mouse model. The neural circuit was activated or inhibited using the chemogenetic method to explore the relationship between the neural circuit and mechanical allodynia and anxiety‐like behaviors. EA combined with the chemogenetic method was used to explore whether the effects of EA were related to this neural circuit.

**Results:**

EA attenuated mechanical allodynia and anxiety‐like behaviors in SNI mice, which may be associated with the activity of CaMKII neurons in the basolateral amygdala (BLA). Inhibition of BLA^CaMKII^‐rACC induced mechanical allodynia and anxiety‐like behaviors in sham mice. Activation of the BLA^CaMKII^‐rACC alleviated neuropathic pain and anxiety‐like behaviors in SNI mice. The analgesic and anxiolytic effects of 2 Hz EA were antagonized by the inhibition of the BLA^CaMKII^‐rACC.

**Conclusion:**

BLA^CaMKII^‐rACC mediates mechanical allodynia and anxiety‐like behaviors. The analgesic and anxiolytic effects of 2 Hz EA may be associated with the BLA^CaMKII^‐rACC.

## Graphical abstract

Schematic diagram illustrating the potential mechanism by which the BLA^CaMKII^‐rACC neural circuit mediates mechanical allodynia and anxiety‐like behaviors in SNI mice, as well as the analgesic and anxiolytic effects of EA.

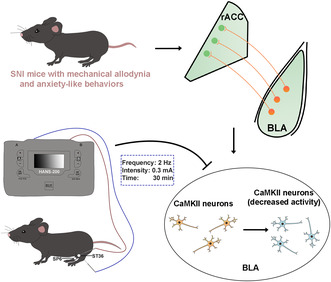

## 1 INTRODUCTION

Chronic pain causes tremendous burdens, affecting over 30% of people around the world.^1^ Patients with chronic pain can suffer from mental disorders, including anxiety.^2^, ^3^ Currently, the use of selective serotonin reuptake inhibitors (SSRIs) and 5‐serotonin noradrenaline reuptake inhibitors (SNRIs) is beneficial in the treatment of chronic neuropathic pain and mental disorders.^4^, ^5^ Meanwhile, SSRIs and SNRIs often produce adverse effects such as gastrointestinal effects.^6^, ^7^ Electroacupuncture (EA), as a safe treatment, can alleviate chronic neuropathic pain and anxiety.^8^, ^9^ However, the mechanism of EA is still not fully elucidated.

The amygdala is essential for regulating pain and anxiety.^10^ Injection of glutamate receptor antagonists into basolateral amygdala (BLA) in mice can alleviate neuropathic pain.^11^ Injection of the antidepressant paroxetine into the BLA of neuropathic pain mice reduces anxiety‐related behaviors.^12^ Our previous research has indicated BLA is linked to anxiety‐like behaviors induced by spared nerve injury (SNI).^13^ Therefore, our study focused on the role of BLA regarding chronic neuropathic pain and anxiety‐like behaviors.

Rostral anterior cingulate cortex (rACC) is involved in neuropathic pain‐related negative emotion.^14^, ^15^ Besides, glutamatergic (Glu) receptors on the rACC are engaged in the emotional response to pain.^16^ Our study showed there are CaMKII neuronal projections from BLA to rACC (BLA^CaMKII^‐rACC).^17^ However, it is currently unclear whether BLA^CaMKII^‐rACC is involved in chronic neuropathic pain and anxiety‐like behaviors.

In our previous research, EA mediated BLA to relieve mechanical allodynia and anxiety‐like behaviors.^13^ EA regulated rACC to suppress negative emotions.^18^ However, it remains unclear whether EA alleviates chronic neuropathic pain and anxiety‐like behaviors via the BLA^CaMKII^‐rACC.

Therefore, this study aims to investigate whether the BLA^CaMKII^‐rACC neural circuit mediates chronic neuropathic pain and anxiety‐like behaviors and whether the analgesic and anxiolytic effects of EA are related to the BLA^CaMKII^‐rACC neural circuit. Chronic neuropathic pain mice prepared by SNI surgery. Activity of CaMKII neurons in BLA detected by immunofluorescence technique. The anatomical connection between BLA and rACC was validated via viral tracing techniques. The activation or inhibition of the BLA^CaMKII^‐rACC neural circuit was manipulated by chemogenetic method. Mechanical allodynia was assessed via paw withdrawal thresholds (PWTs). Both elevated plus maze test (EPMT) and open field test (OFT) were used to observe anxiety‐like behaviors.

## 2 MATERIALS AND METHODS

### 2.1 Animals

All experiments mice were adult male C57BL/6J mice (22–25 g, 8–10 w). The mice were provided and raised by the Experimental Animal Center of Zhejiang Chinese Medical University. Ventilation and air filtration units were available for the study. The mice were raised under a 12‐h light/dark cycle (light cycle 8:00 a.m.–8:00 p.m.) at room temperature of 23–25°C and humidity of 40%–60%. Each cage housed 4 male mice with corn cob bedding at the bottom. The mice were fed with standard pellet chow and water *ad libitum*. The experimental operations complied with the experimental ethics requirements of the Experimental Animal Management and Ethics Committee of Zhejiang Chinese Medical University (IACUC‐20210118‐07) and were conducted following the relevant provisions of the Regulations on the Administration of Laboratory Animals of the People's Republic of China and the spirit of humanitarianism.

### 2.2 von Frey filament test

The mice were placed on wire mesh and covered with translucent plexiglass cover. They were allowed to acclimate to the environment for 1 h. After the mice were quiet, the mice were stimulated with von Frey filaments in the plantar surface of the left hind paw until the von Frey filaments bent into an S‐shape and held for 6–8 s. A positive reaction was counted when mice retracted, licked, or flinched their claws quickly. Stimulation intervals for von Frey filaments should be at least 1 min each time. The force of the von Frey filament with three positive reactions out of five tests was recorded as PWTs.^19^ PWTs were measured at baseline, day 7, and day 14 after modeling.^20^

### 2.3 Chronic neuropathic pain mouse model

SNI surgery was used to establish a chronic neuropathic pain model.^21^ Mice were anesthetized with 0.3% pentobarbital sodium (60 mg/kg, ip). The hair of the left hind limb was removed. The skin was disinfected with iodophor and 75% ethanol. An incision was made on the skin above the midpoint between the tibial head and the greater trochanter of the femur. Then, the muscles were bluntly dissected to expose the sciatic nerve. The peroneal nerve and common peroneal nerve were tightly ligated with non‐absorbent 6–0 sutures and the nerves were transected. A section of 2–3 mm was removed distal to the ligature, leaving the tibial nerve intact. The incision was closed in layers and disinfected with iodophor. The sham group underwent the same surgery without the ligation or severance of the nerve.

### 2.4 Viral injection

Mice were anesthetized with 0.3% sodium pentobarbital (60 mg/kg, ip). The mice were fixed on a stereotaxic frame (RWD, 68025, Shenzhen, China). A glass microelectrode for injection was connected to an infusion pump (WPI, UMC4, Sarasota, FL, United States). A volume of 160 nL of virus was administered into the BLA at a rate of 100 nL/min (BLA: anterior–posterior: −1.36 mm; mediolateral: ±3.20 mm; dorsoventral: −4.25 mm). A volume of 90 nL of virus was injected into the rACC at a rate of 60 nL/min (rACC: anterior–posterior: +1.35 mm; mediolateral: ±0.25 mm; dorsoventral: −0.85 mm). A dental drill (WPI, OmniDrill35, Sarasota, FL, United States) was used to create the cranial openings. At the end of the infusion, the glass microelectrode was left at the injection site for 10 min to prevent the virus from overflowing. The BLA coordinates and rACC coordinates were determined based on Paxinos and Franklin's The Mouse Brain in Stereotaxic Coordinates (Fourth version).

### 2.5 Tracer virus injection strategy

Mice were injected with anterograde tracer virus (AAV2/9‐CaMKIIα‐EGFP, 5.34 × 10^12^ vg/mL, BrainVTA, China, PT‐0290) in the right BLA. The virus (AAV2/R‐CaMKIIα‐EGFP, 5.24 × 10^12^ vg/mL, BrainVTA, PT‐0290) was injected into the right rACC for retrograde monosynaptic tracking. After 2 weeks, the tracer virus successfully transfected neurons in BLA and rACC. Then, the mice were anesthetized with 0.3% sodium pentobarbital (60 mg/kg, ip) and perfused sequentially with saline (20 mL) and 4% paraformaldehyde (20 mL) via the cardiac. Finally, 20 μm brain slices were prepared for recording green fluorescent protein signals.

### 2.6 Chemogenetic method

The BLA^CaMKII^‐rACC neural circuit was manipulated via the chemogenetic method to determine whether the neural circuit modulated mechanical allodynia and anxiety‐like behaviors. The mice were injected rAAV2/9‐CaMKIIα‐DIO‐hM4Di‐mCherry‐WPRE‐pA (3.38 × 10^12^ vg/mL, BrainVTA, China, PT‐1143), rAAV2/9‐CaMKIIα‐DIO‐hM3Dq‐mCherry‐WPRE‐pA (3.04 × 10^12^ vg/mL, BrainVTA, China, PT‐1144) or rAAV2/9‐CaMKIIα‐DIO‐mCherry‐WPRE‐pA (5.35 × 10^12^ vg/mL, BrainVTA, China, PT‐1167) into the BLA. rAAV2/R‐CaMKIIα‐Cre (6.65 × 10^12^ vg/mL, BrainVTA, China, PT‐0220) was injected into the rACC. Clozapine‐N‐oxide (CNO) (0.2 mg/mL, dissolved in dimethyl sulfoxide and diluted with 0.9% saline, 2 mg/kg, ip) was intraperitoneally injected on days 8, 10, 12, 14, and 16 after SNI/sham surgery. Among them, rAAV2/9‐CaMKIIα‐DIO‐hM4Di‐mCherry‐WPRE‐pA and rAAV2/R‐CaMKIIα‐Cre were injected bilaterally to inhibit the BLA^CaMKII^‐rACC neural circuit. rAAV2/9‐CaMKIIα‐DIO‐hM3Dq‐mCherry‐WPRE‐pA and rAAV2/R‐CaMKIIα‐Cre were injected into the right side to activate the BLA^CaMKII^‐rACC neural circuit. The rAAV2/9‐CaMKIIα‐DIO‐mCherry‐WPRE‐pA is a control virus without any activating or inhibitory effect.

### 2.7 EA treatment

EA treatment was conducted on the 8th, 10th, 12th, 14th, and 16th days after SNI surgery. In addition to the EA group, the sham, SNI, and SNI‐sham EA groups were all fixed loosely. Bilateral Zusanli (ST36) and Sanyinjiao (SP6) acupoints were selected. 0.16 × 7 mm acupuncture needles were directly inserted into the acupoints for 5 mm in the EA group. The two needles on the same side were connected to the Hans acupoint nerve stimulator (HANS‐200A, Beijing Hua Wei Industrial Development Co). The intensity of EA was 0.3 mA, the frequency was 2 Hz and the stimulation time was 30 min. In the SNI‐sham EA group, electrodes, but no current, were attached to the acupuncture needles.

### 2.8 Elevated plus maze test (EPMT)

The EPMT is the most commonly used test for studying general anxiety‐like behavior.^22^ Mice were subjected to EPMT on day 14 after sham/SNI surgery. The day before EPMT, the mice were transferred to the behavioral room to acclimatize. The room temperature was 23–25°C and the humidity was 40%–60%. The elevated plus maze (EPM) was composed of two open arms (30 × 6 cm), two closed arms (30 × 6 × 15 cm), and a center area (6 × 6 cm) with a height of 35 cm. The open arms and closed arms are crossed vertically. In the beginning, each mouse was placed in the center area with its head facing the open arm. After the experiment, the urine and feces of the mice were cleaned up. To remove the odor left by the last animal, the EPM will be cleaned with 75% ethanol and double‐distilled water. The mice's behaviors during the test were recorded on a video tracking system (ANY‐maze V6.14, Stoelting, USA). The video was recorded for 5 min and 30 s, with the first 30 s being the acclimatization phase.

### 2.9 Open field test (OFT)

The OFT was conducted on day 16 after surgery to determine whether mice displayed anxiety‐like behaviors.^23^ The day before OFT, the mice were relocated to the behavioral room. The open field (OF) was a 40 × 40 × 40 cm^3^ uncovered cube. The bottom of the cube was divided equally into 16 squares of identical area. The outer 12 square areas were defined as the peripheral area. The 4 square areas in the middle are defined as the central zone (with a total area of 20 × 20 cm^2^). The mice were placed in the center zone at the beginning. Finally, the mice's urine and feces were cleaned up. The interior of the OF was cleaned with 75% ethanol and double‐distilled water in turn. The mice's behaviors during testing were recorded on a video tracking system (ANY‐maze V6.14, Stoelting, USA). The duration of the video is 5 min and 30 s.

### 2.10 Immunofluorescence staining

Approximately 90 min after OFT, mice were deeply anesthetized with sodium pentobarbital (60 mg/kg, ip). The mice were perfused transcardially with 0.9% saline and 4% (w/v) paraformaldehyde sequentially. After perfusion, the mice brains were removed and placed in 4% (w/v) paraformaldehyde for 24 h and then subjected to gradient dehydration with 15% and 30% (w/v) sucrose. The brains were sliced into frozen coronal sections of 20 μm thickness using the freezing microtome (CryoStar NX50 HOP, Thermo Fisher Scientific).

The slices were mounted on gelatin‐coated glass. The slices were rewarmed at 37°C for 1 h, and they were washed with Tris Buffered Saline with Tween‐20 (TBST) on a shaker 6 times for 10 min each. The slices were blocked with 10% donkey serum (with 0.3% Triton X‐100) for 1 h at 37°C and incubated with the primary antibodies for 20 h at 4°C. The primary antibodies included anti‐CaMKII (mouse, 1:200, Abcam, ab22609) and anti‐cFos (rabbit, 1:500, Abcam, ab190289). The slices were then rewarmed again at 37°C for 1 h, washed 6 times, and incubated with Alexa Fluor 647‐conjugated secondary antibody (1:1000, Abcam, ab150111) and Alexa Fluor 488‐conjugated secondary antibody (1:800, Jackson ImmunoResearch Labs, 711‐545‐152) for 1 h at 37°C. The slices were washed again with TBST, followed by incubation with 4′,6‐diamidino‐2‐phenylindole (DAPI, Abcam, USA). The fluorescence signal was then observed using a ZEISS digital scanner (ApoTome.2, ZEISS, Germany).

### 2.11 Statistical analysis

All analyses and graphs were calculated and plotted using GraphPad Prism 9. All results are expressed as mean ± standard errors of the mean (SEM). The normality of the distribution of continuous variables was assessed using the Shapiro–Wilk normality test. The PWTs between different groups were tested with two‐way repeated‐measures Analysis of Variance (ANOVA) with Tukey's post‐hoc test. For normally distributed data, Student's *t*‐test (two‐tailed) and one‐way ANOVA followed by Tukey's post‐hoc test were used to compare means of two and multiple groups, respectively. Additionally, the Mann–Whitney *U*‐test and Kruskal–Wallis were employed to compare two or more groups of non‐normally distributed data, respectively. A significance level of *p* < 0.05 was considered statistically significant.

## 3 RESULTS

### 3.1 2 Hz EA reduced mechanical allodynia and anxiety‐like behaviors in SNI mice

**FIGURE 1:**
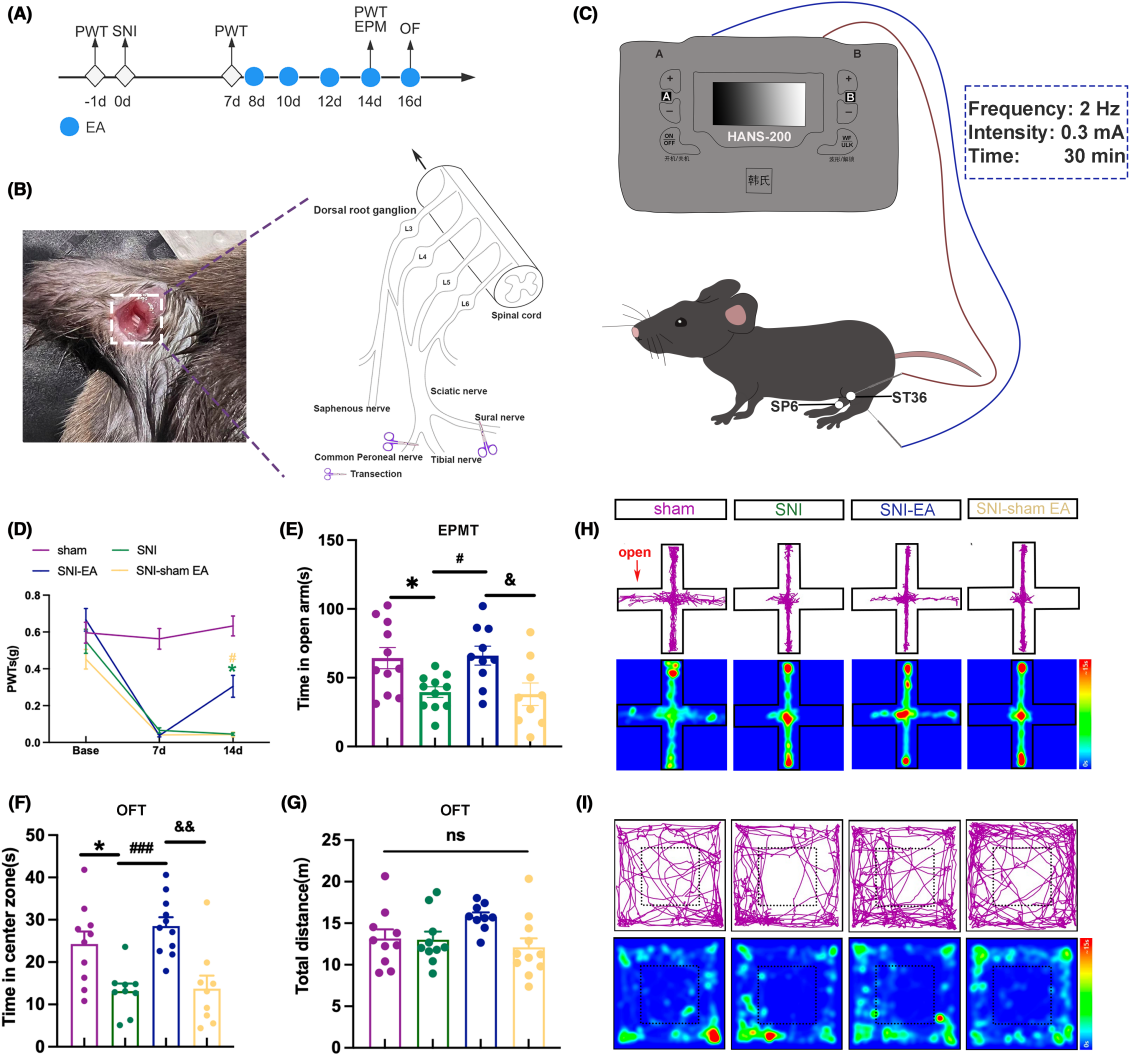
2 Hz EA reduced mechanical allodynia and anxiety‐like behaviors in SNI mice. (A) Experimental flow chart. (B) SNI surgery schematic diagram. (C) Schematic diagram of EA intervention. The acupoints for EA intervention were bilateral Zusanli (ST36) and Sanyinjiao (SP6), with a stimulation frequency of 2 Hz, intensity of 0.3 mA, and 30 min. (D) A comparison of PWTs among 4 groups. (E) Time spent in the open arms among 4 groups. (F) Time spent in the center zone among 4 groups. (G) The total distance traveled by the 4 groups. (H) Representative motion trajectories and activity heat maps in EPMT (I) Representative motion trajectories and activity heat maps in OFT. *p < 0.05, ^###^ *p* < 0.001 compared with SNI group; ^#^ *p* < 0.05, ^&&^ *p* < 0.01 compared with SNI‐EA group; ^&^ *p* < 0.05 compared with SNI‐sham EA group; ns, not significant. *n* = 8–12 (D–G). (D) two‐way ANOVA and Tukey's test; (E–G) One‐way ANOVA and Tukey's test.

Research findings indicate that EA effectively alleviates mechanical allodynia and anxiety‐like behaviors.^24^, ^25^, ^26^ Therefore, we aim to further investigate the effects of 2 Hz EA on mechanical allodynia and anxiety‐like behaviors in neuropathic pain mice (Figure 1A). We established a chronic neuropathic pain mouse model by employing SNI injury on C57BL/6J mice (Figure 1B), following previously reported protocols.^27^ Similar to previous studies, we chose bilateral Zusanli (ST36) and Sanyinjiao (SP6) as the sites for EA stimulation (Figure 1C).^25^, ^26^

Compared to the sham mice, the PWTs in the SNI mice decreased (Figure 1D). The SNI mice spent less time in the open arm and central zone (Figure 1E,F). The result suggests that SNI surgery can induce neuropathic pain and anxiety‐like behaviors in mice.

After EA intervention, the PWTs of the SNI‐EA mice increased compared to SNI mice and SNI‐sham EA mice (Figure 1D). SNI‐EA mice exhibited an increase in open‐arm and central zone time (Figure 1E,F). The results suggest that 2 Hz EA is effective at reducing mechanical allodynia and anxiety‐like behaviors in SNI mice. At the same time, there were no statistically significant differences in the total distance traveled among the 4 groups (Figure 1G), which suggests the locomotor activity of the 4 groups was not affected. Figure 1H,I show the trajectory maps and heat maps of the 4 groups. These results underscore the efficacy of the SNI mice in inducing mechanical allodynia and anxiety‐like behaviors. Moreover, 2 Hz EA significantly mitigated chronic neuropathic pain and anxiety‐like behaviors in SNI mice.

### 3.2 The effects of EA on mechanical allodynia and anxiety in SNI mice may be linked to BLA CaMKII neurons activity

Research suggests BLA mediates neuropathic pain.^11^, ^28^, ^29^ Our preliminary research indicates BLA is also associated with anxiety‐like behavior induced by SNI.^13^ Modulating BLA influences anxiety‐like behaviors in neuropathic pain mice.^10^ Therefore, we aim to further explore whether the analgesic and anti‐anxiety effects of EA on SNI mice are associated with the BLA.

**FIGURE 2:**
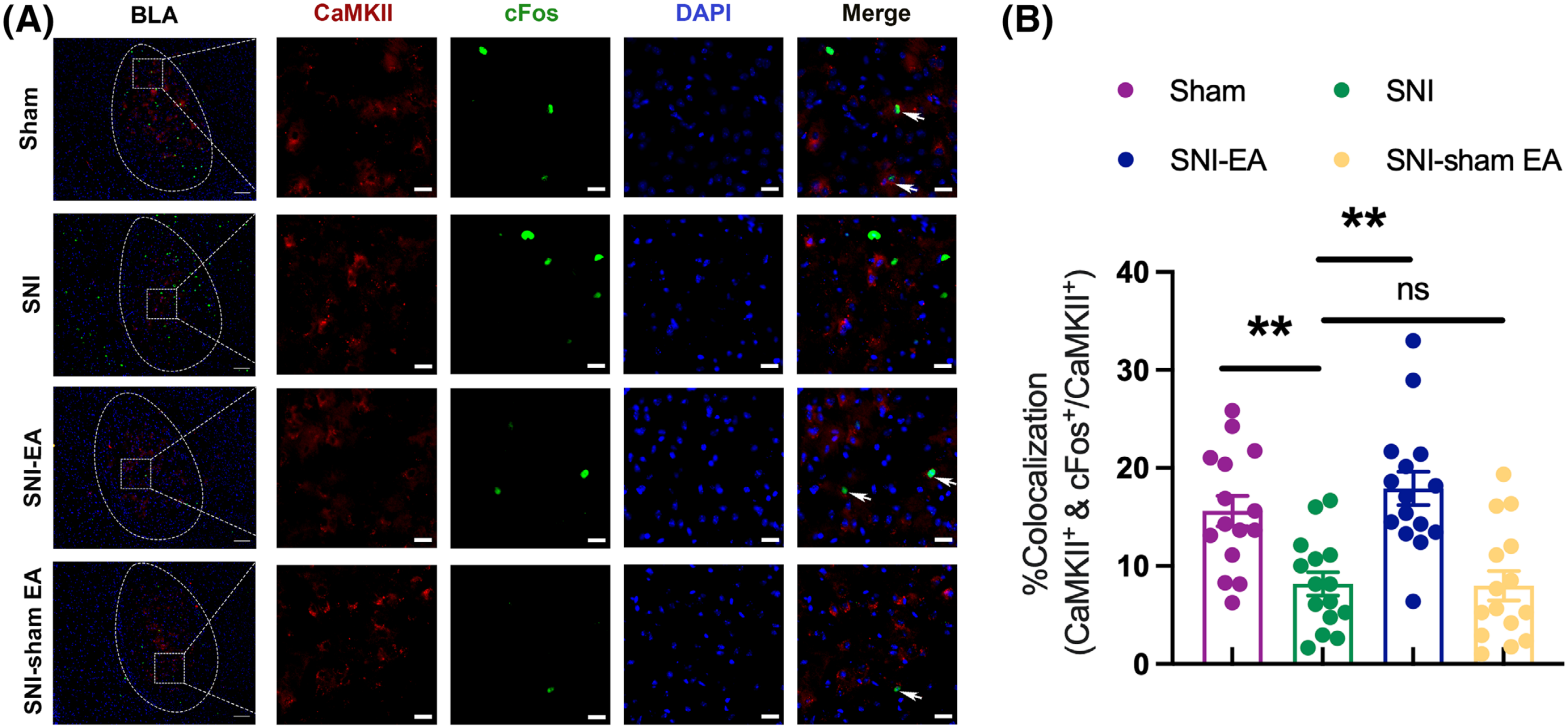
The colocalization of CaMKII and cFos in the BLA. (A) The representative images of the colocalization of CaMKII and cFos in the BLA among 4 groups. The red fluorescence represents CaMKII, the green fluorescence indicates the neuronal activation marker protein cFos and the blue fluorescence represents the nuclear counterstain DAPI. Scale bar: 20 μm. (B) The statistical results of the colocalization counts of CaMKII and cFos in the BLA. **p < 0.01, compared with SNI group; ns, not significant. *n* = 3 (with 5 brain slices per mouse).

cFos is a marker for neuronal activity. When neurons are activated, the expression of cFos is upregulated.^30^ As shown in Figure 2A,B, compared to the sham group, the co‐expression rate of cFos in BLA CaMKII neurons is reduced in SNI mice, suggesting that the mechanical allodynia and anxiety‐like behavior in SNI mice may be associated with decreased activity of BLA CaMKII neurons. Meanwhile, compared to the SNI group, the co‐expression rate of cFos in BLA CaMKII neurons is higher in the SNI‐EA group, indicating that the analgesic and anti‐anxiety effects of EA may be linked to the activity of BLA CaMKII neurons.

### 3.3 CaMKII neurons project from BLA to rACC

**FIGURE 3:**
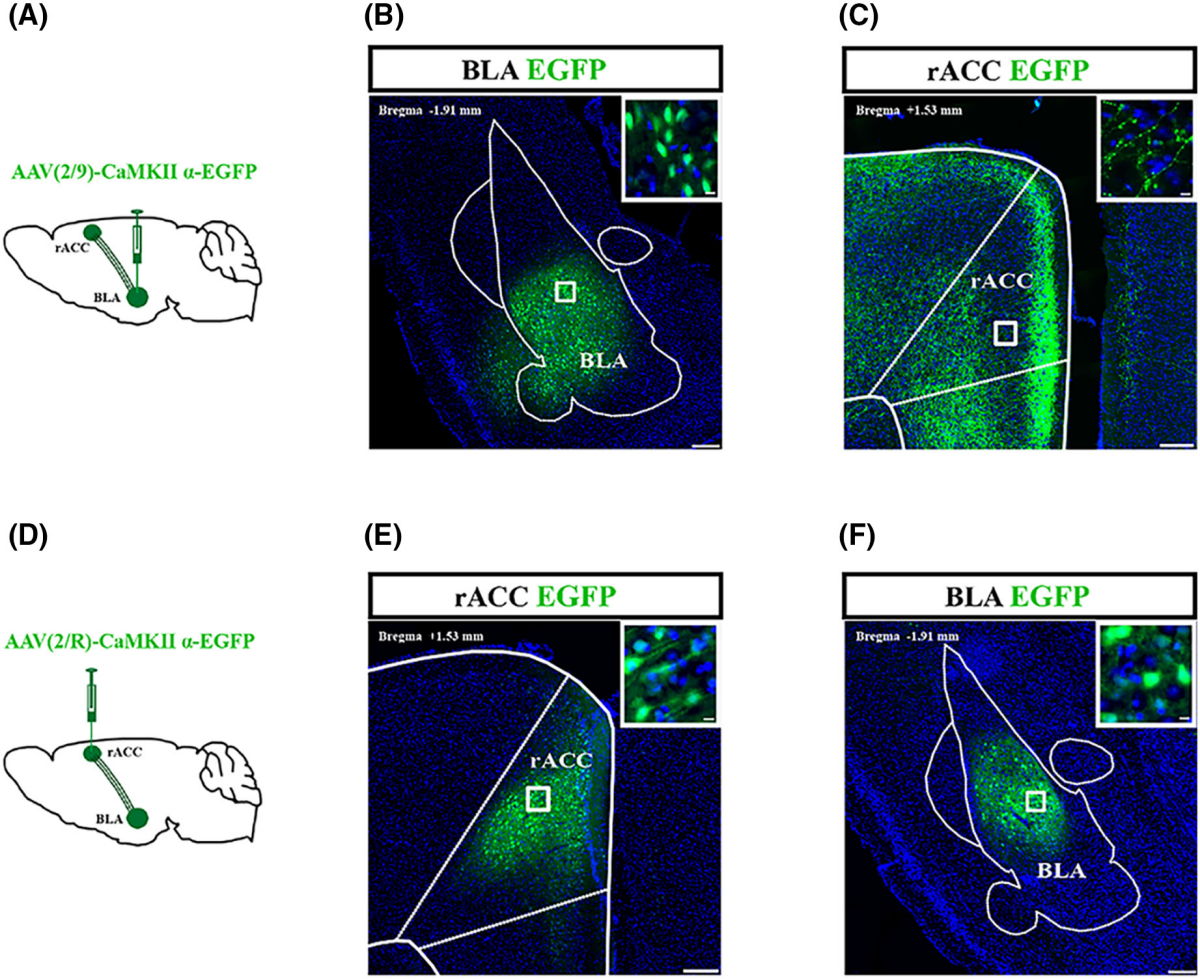
Circuit diagram for the BLA^CaMKII^‐rACC. (A) Schematic diagram of AAV2/9‐CaMKIIα‐EGFP injection in the right BLA. (B) Representative images of right BLA CaMKII neurons projecting to the right rACC in the right BLA and local magnification of the injection site (top right). Scale bar = 200 μm, scale bar =10 μm (top right). (C) Representative images of right rACC fibers projecting from the right BLA. Scale bar = 100 μm (left), scale bar = 10 μm (top right). (D) Schematic diagram of AAV2/R‐CaMKIIα‐EGFP injection in the right rACC. (E) Representative images of the retrograde tracer virus injection site in the right rACC. Scale bar = 100 μm, scale bar = 10 μm (top right). (F) Representative images of the CaMKII neurons in the right BLA retrograding from the right rACC. Scale bar = 200 μm, scale bar = 10 μm (top right).

To investigate whether BLA is structurally related to rACC, an anterograde tracing virus (AAV2/9‐CaMKIIα‐EGFP) was injected into the right BLA (Figure 3A). After 2 weeks, CaMKII neurons that were transfected and labeled by the virus were observed in the right BLA (Figure 3B). In addition, nerve fibers transfected and labeled by the virus could be seen in the right rACC (Figure 3C). Furthermore, we injected retrograde tracer viruses (AAV2/R‐CaMKIIα‐EGFP) into the right rACC (Figure 3D). Two weeks after injection, transfected CaMKII neurons could be visualized in the right rACC (Figure 3E). The CaMKII neurons transfected by the virus could be observed in the right BLA (Figure 3F). Therefore, CaMKII neurons in the BLA project to the rACC.

### 3.4 Inhibition of BLA^CaMKII^ ‐rACC induced mechanical allodynia and anxiety‐like behaviors in sham mice

Our previous research demonstrated the correlation between the activity of BLA CaMKII neurons and chronic neuropathic pain and anxiety‐like behaviors and the existence of the BLA^CaMKII^‐rACC. In addition, rACC is also implicated in chronic neuropathic pain and pain‐related negative emotions.^31^, ^32^ However, the role of the BLA^CaMKII^‐rACC in chronic neuropathic pain and anxiety‐like behaviors remains unclear. Therefore, we used chemogenetic methods in sham mice to manipulate the BLA^CaMKII^‐rACC neural circuit. We observed alterations in PWTs, open arm time, and central time to investigate if BLA^CaMKII^‐rACC plays a role in mechanical allodynia and anxiety‐like behaviors.

**FIGURE 4:**
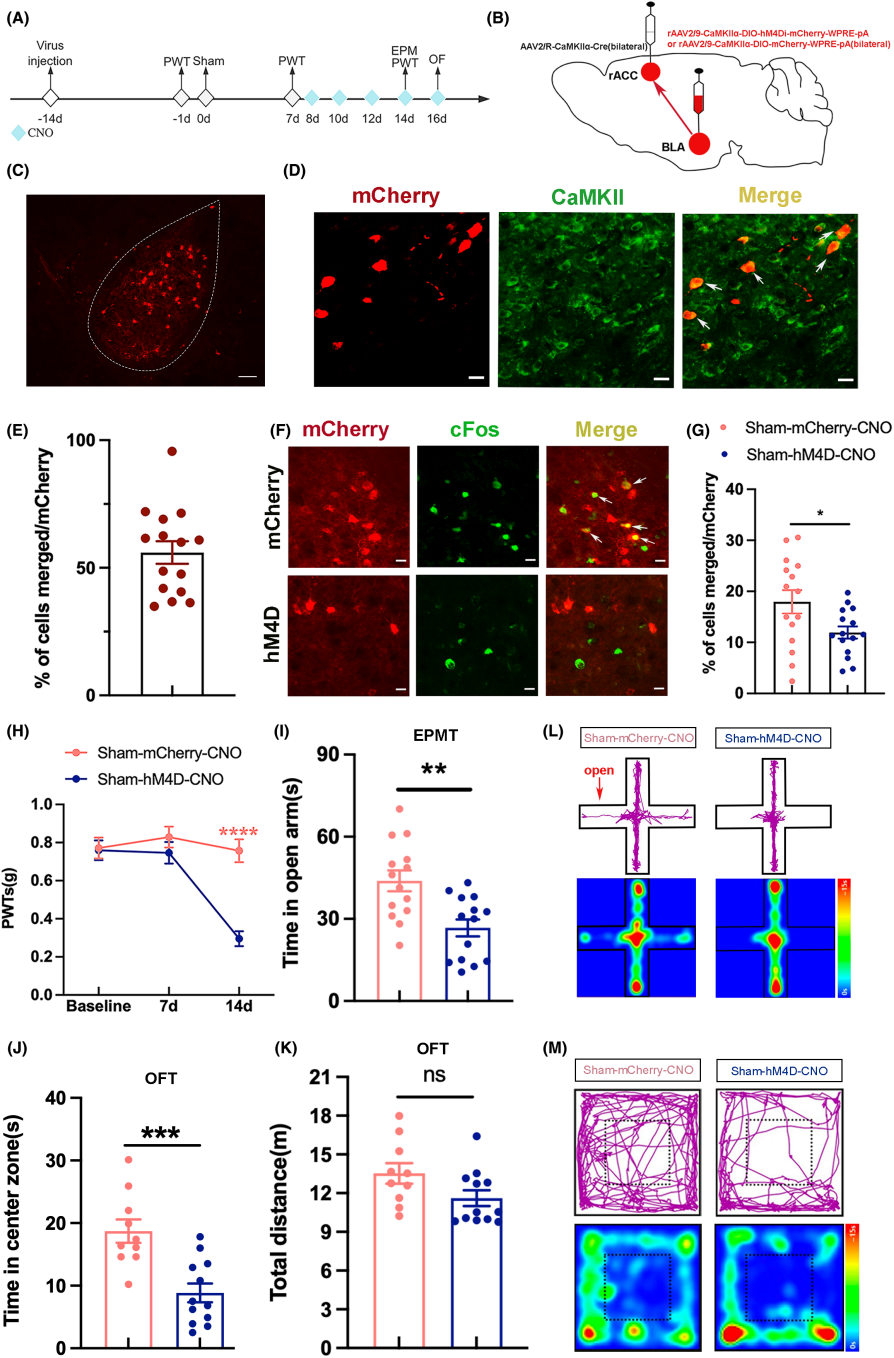
Inhibition of BLA^CaMKII^‐rACC‐induced mechanical allodynia and anxiety‐like behaviors in sham mice. (A) Experimental flow chart. (B) Chemogenetic virus injection diagram. (C) Image of viral expression on the BLA. Scale bar = 100 μm. (D) Representative images of BLA CaMKII neurons infected by DIO‐mCherry (red) and stained with CaMKII (green). Scale bar = 20 μm. (E) The percentage of colocalization of mCherry and CaMKII (*n* = 15 slices from 3 mice). (F) Representative images of BLA CaMKII neurons infected by DIO‐mCherry (red) and stained with cFos (green). Scale bar = 20 μm. (G) The percentage of colocalization of mCherry and cFos (*n* = 15 slices from 3 mice, *p < 0.05, unpaired sample *t*‐test). (H) The comparison of PWTs. (I) Time spent in the open arms. (J) Time spent in the center zone. (K) The total distance traveled by the 2 groups in the OFT did not differ statistically. (L) Representative motion trajectories and activity heat maps in EPMT. (M) Representative motion trajectories and activity heat maps in OFT. ****p < 0.0001, **p < 0.01, ***p < 0.001, compared with sham‐mCherry‐CNO group; ns, not significant. *n* = 10–15 (H–K). (H) two‐way ANOVA and Tukey's test; (I–K) unpaired‐sample *t*‐test.

In sham‐hM4D‐CNO group, bilateral injections of rAAV2/9‐CaMKIIα‐DIO‐hM4Di‐mCherry‐WPRE‐pA were administered into the BLA, while bilateral injections of rAAV2/R‐CaMKIIα‐Cre were delivered into the rACC to inhibit bilateral BLA^CaMKII^‐rACC. In sham‐mcherry‐CNO group, the BLA was injected bilaterally with AAV2/9‐CaMKIIα‐DIO‐mCherry‐WPRE‐pA, the rACC was injected bilaterally with rAAV2/R‐CaMKIIα‐Cre to be used as a control group (Figure 4A,B). After 2 weeks, viral expression was observed in BLA (Figure 4C). Approximately 56.0% of the BLA‐projecting neurons labeled with mCherry were immunoreactive for CaMKII (Figure 4D,E). To determine whether the hM4D virus inhibits the activity of BLA CaMKII neurons projecting to rACC, we compared the percentage of colocalization of the virus‐labeled neurons with cFos between the sham‐mCherry‐CNO and sham‐hM4D‐CNO groups (Figure 4F). Results showed that the hM4D virus reduced the activity of BLA CaMKII neurons projecting to rACC from 17.9% to 11.9% (Figure 4G).

In comparison to the sham‐mCherry‐CNO group, the sham‐hM4D‐CNO group exhibited significantly lower PWTs (Figure 4H), suggesting that inhibition of BLA^CaMKII^‐rACC induced mechanical allodynia in sham mice. Compared to the sham‐mCherry‐CNO group, the sham‐hM4D‐CNO group spent less time in the open arm and the center zone (Figure 4I,J). There was no notable variance observed in the total distance traveled (Figure 4K), suggesting similar locomotor abilities between the 2 groups. Figure 4L,M depict the representative motion trajectories and heat maps in EMPT and OFT, respectively. Hence, the results suggest inhibition of BLA^CaMKII^‐rACC‐induced mechanical allodynia and anxiety‐like behaviors in sham mice.

### 3.5 Activation of BLA^CaMKII^ ‐rACC attenuated mechanical allodynia and anxiety‐like behaviors in SNI mice

**FIGURE 5:**
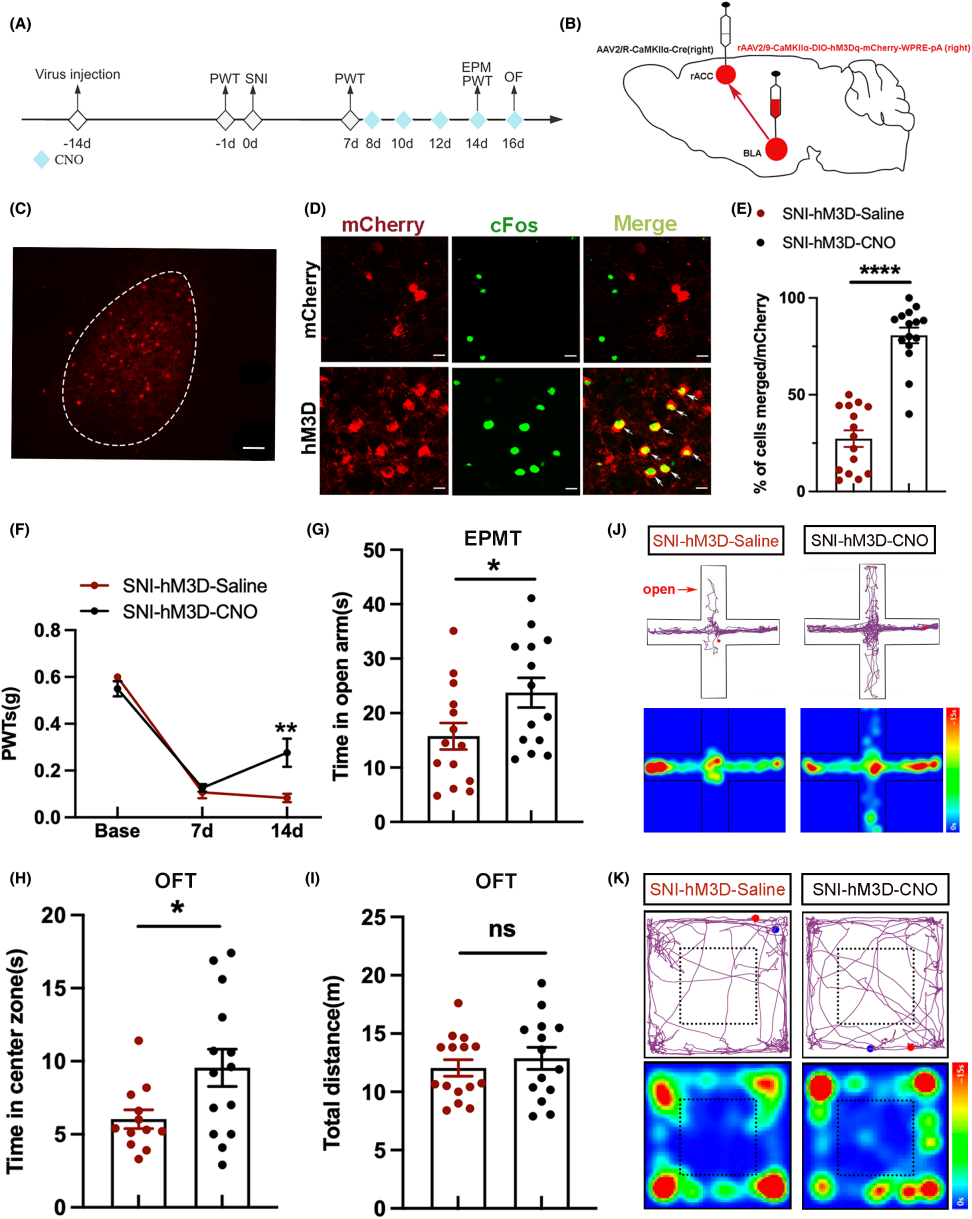
Activation of the BLA^CaMKII^‐rACC alleviated mechanical allodynia and anxiety‐like behaviors in SNI mice. (A) Experimental flow chart. (B) Chemogenetic virus injection diagram. (C) Representative image of neurons in the BLA transfected and labeled with mCherry virus. Scale bar = 100 μm. (D) Representative images of BLA CaMKII neurons infected by DIO‐mCherry (red) and stained with cFos (green). Scale bar = 20 μm. (E) The percentage colocalization of mCherry and cFos (*n* = 15 slices from 3 mice, ****p < 0.0001, unpaired sample *t*‐test). (F) A comparison of PWTs. (G) Time spent in the open arms. (H) Time spent in the center zone. (I) The total distance traveled by the 2 groups. (J) Representative motion trajectories and activity heat maps in EPMT. (K) Representative motion trajectories and activity heat maps in OFT. *p < 0.05, **p < 0.01, compared with the SNI‐hM3D‐Saline group; ns, not significant. *n* = 8 (F). *n* = 12–15 (G–I). (F) two‐way ANOVA and Tukey's test, (G–I) unpaired‐sample *t*‐test.

Since the previous result shows the inhibition of BLA^CaMKII^‐rACC in sham mice can induce mechanical allodynia and anxiety‐like behaviors, we next investigated whether mechanical allodynia and anxiety‐like behaviors in SNI mice could be attenuated by activation of BLA^CaMKII^‐rACC (Figure 5A). First, rAAV2/9‐CaMKIIα‐DIO‐hM3Dq‐mCherry‐WPRE‐pA was injected into the right BLA, and AAV‐CaMKIIα‐Cre was injected into the right rACC to activate BLA^CaMKII^‐rACC (Figure 5B,C). Next, we examined the colocalization of CaMKII neurons with cFos (Figure 5D). It turned out that BLA CaMKII neurons projecting to rACC were significantly activated by the hM3D virus (Figure 5E).

Compared to the SNI‐hM3D‐Saline group, the PWTs of the SNI‐hM3D‐CNO group were higher (Figure 5F). The PWTs results suggest activation of BLA^CaMKII^‐rACC relieves mechanical allodynia in SNI mice. In addition, SNI‐3D‐CNO mice spent more time in the open arm and center zone compared with the SNI‐hM3D‐Saline group (Figure 5G,H). The total distance was not statistically different between the 2 groups (Figure 5I). Motion trajectory maps and heat maps of the 2 groups in EPMT and OFT are depicted in Figure 5J,K, respectively. Therefore, it can be concluded that activation of BLA^CaMKII^‐rACC can mitigate mechanical allodynia and anxiety‐like behaviors in SNI mice.

### 3.6 Inhibition of BLA^CaMKII^ ‐rACC antagonized the analgesic and anti‐anxiety effects of EA on SNI mice

The above results indicate that EA can alleviate mechanical allodynia and anxiety‐like behaviors in SNI mice, possibly associated with the activity of BLA CaMKII neurons. Besides, BLA^CaMKII^‐rACC is involved in the mechanical allodynia and anxiety‐like behaviors of both sham and SNI mice. However, it remains unclear if the analgesic and anti‐anxiety effects of EA on SNI mice are linked to BLA^CaMKII^‐rACC.

**FIGURE 6:**
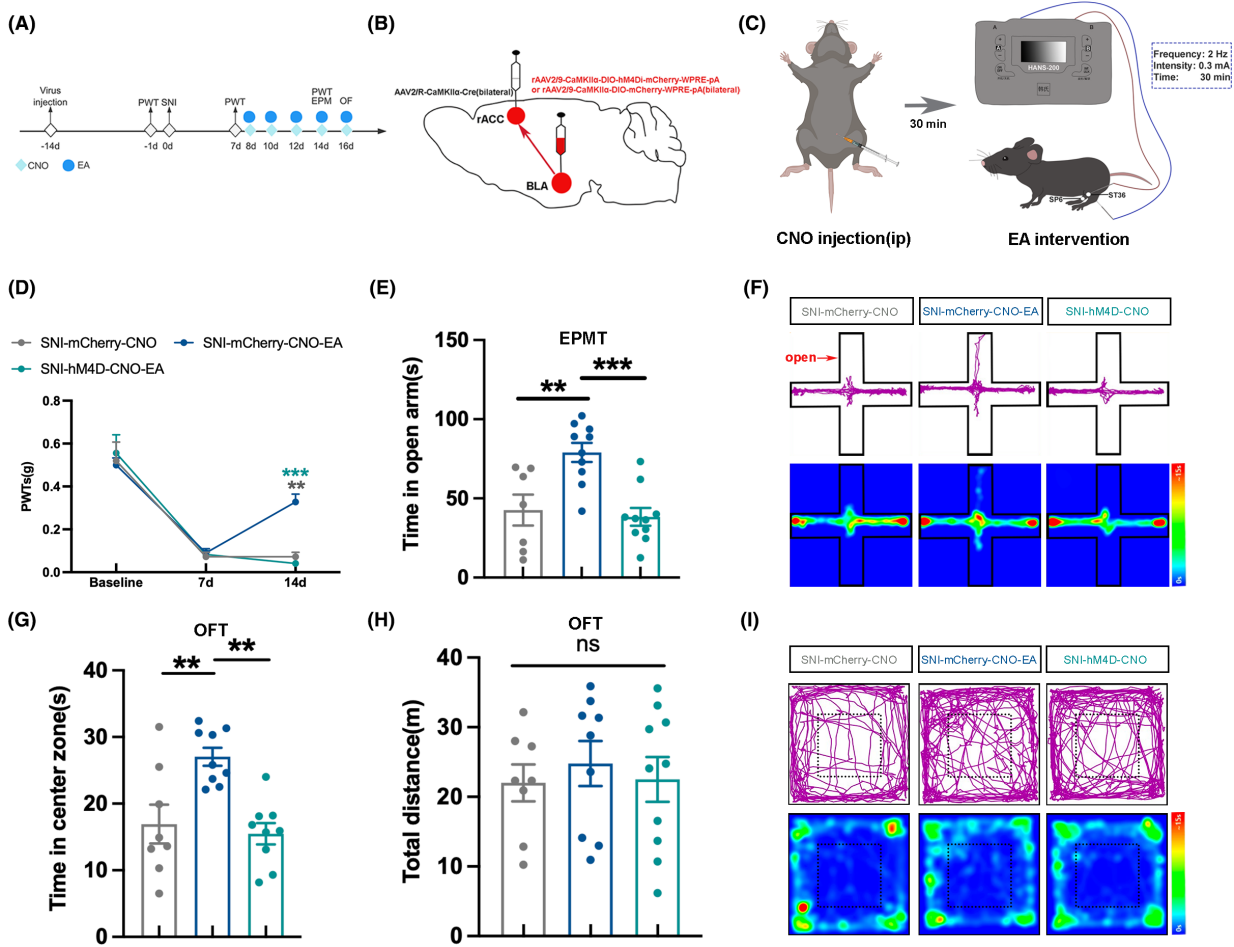
Analgesic and anxiolytic effects of EA were associated with BLACaMKII‐rACC neural circuit. (A) Experimental flow chart. (B) Chemogenetic virus injection diagram. (C) After intraperitoneal injection of CNO for 30 min, EA intervention was conducted on SNI mice at the acupoints Zusanli (ST36) and Sanyinjiao (SP6) bilaterally. The EA had a frequency of 2 Hz, an intensity of 0.3 mA, and 30 min. (D) A comparison of PWTs. (E) Time spent in the open arm. (F) Time spent in the center zone. (G) The total distance covered by the 3 groups in the OFT showed no statistically significant difference. (H) Representative motion trajectories and activity heat maps in EPMT. (I) Representative motion trajectories and activity heat maps in OFT. Compared to the SNI‐mCherry‐CNO‐EA group, **p < 0.01, ***p < 0.001; ns, not significant. *n* = 7–10 (C–F). (C) two‐way ANOVA and Tukey's test. (D–F) one‐way ANOVA and Tukey's test.

To investigate whether the effects of EA are mediated through the BLA^CaMKII^‐rACC. In the SNI‐hM4D‐CNO‐EA group, bilateral BLA was injected with rAAV2/9‐CaMKIIα‐DIO‐hM4Di‐mCherry‐WPRE‐pA, bilateral rACC was injected with rAAV2/R‐CaMKIIα‐Cre to inhibit the BLA^CaMKII^‐rACC. After administering CNO injection to SNI mice, EA intervention was performed 30 min later (Figure 6A,B), which allowed us to observe whether the effects of EA were influenced when the BLA^CaMKII^‐rACC was inhibited (Figure 6A,B).

Compared to the SNI‐mCherry‐CNO group, mice in the SNI‐mCherry‐CNO‐EA group showed higher PWTs (Figure 6C), confirming the analgesic effect of EA. In comparison to the SNI‐mCherry‐CNO‐EA group, the SNI‐hM4D‐CNO‐EA group exhibited notably lower PWTs (Figure 6C). The PWTs result indicates the analgesic effect of EA is antagonized when the BLA^CaMKII^‐rACC is inhibited.

Compared to the SNI‐mCherry‐CNO group, the SNI‐mCherry‐CNO‐EA group spent more time in the open arm and center zone (Figure 6D,E). The SNI‐hM4D‐CNO‐EA group spent less time in both the open arm and center zone compared to the SNI‐mCherry‐CNO‐EA group (Figure 6D,E). Meanwhile, the total distance traveled by the 3 groups was not statistically different (Figure 6F). Figure 6G,H show the trajectory map and heat map of the 3 groups in the EPMT and OFT, respectively.

Therefore, the above results indicate that when the BLA^CaMKII^‐rACC neural circuit is inhibited, the analgesic and anti‐anxiety effects of EA are antagonized. This suggests that the BLA^CaMKII^‐rACC is involved in the analgesic and anti‐anxiety mechanism of EA.

## 4 DISCUSSION

Chronic pain is a major cause of disability globally, placing a huge burden on society and families.^33^ Patients with chronic pain tend to show higher features of co‐morbidity with psychiatric disorders, including anxiety.^34^ Clinical studies have found that antidepressant medications are effective in relieving negative emotions but not pain, often causing side effects.^35^, ^36^, ^37^, ^38^, ^39^ Therefore, it is crucial to further explore the potential mechanisms of pain‐related negative emotion. Our previous study found that EA had a significant alleviating effect on chronic pain and pain‐related negative emotions.^40^ So we would like to further explore the potential mechanisms of EA intervention for pain and pain‐related negative emotions.

Acupuncture is a quite safe treatment.^41^, ^42^ Our previous study found that 2 Hz EA of bilateral Zusanli (ST36) and Sanyinjiao (SP6) alleviated the chronic neuropathic pain and anxiety‐like behaviors,^20^ so we did not further screen the frequency and acupoints for EA in this study. In this study, we validated the analgesic and anxiolytic effects of 2 Hz EA on SNI mice. However, the mechanisms underlying the analgesic and anxiolytic effects of EA are still unclear.

The amygdala plays an important role in pain and anxiety regulation.^43^, ^44^, ^45^ As part of the amygdala, the BLA is involved in both hyperalgesia and anxiety.^46^, ^47^, ^48^, ^49^, ^50^ In our previous study, it was observed that BLA mediates anxiety‐like behaviors related to pain.^13^ Glutamate receptor antagonist was found to exert a powerful analgesic effect in BLA.^11^ Our study also found the activity of BLA CaMKII neurons was reduced after SNI surgery. It is suggested that mechanical allodynia and anxiety‐like behaviors in SNI mice may be related to the activity of BLA CaMKII neurons.

Moreover, after EA intervention in SNI mice, mechanical allodynia and anxiety‐like behaviors were alleviated, accompanied by an elevation in the activity of BLA CaMKII neurons. Hence, our study focuses on the correlation between the activity of BLA CaMKII neurons and the manifestation of mechanical allodynia and anxiety‐like behaviors.

rACC is crucial to the regulation of emotions.^51^, ^52^ Activation of N‐methyl‐D‐aspartate receptors in the rACC is required to acquire pain‐related negative emotions.^31^ Activation of N‐methyl‐D‐aspartate receptors on rACC inhibits emotional responses to pain.^16^, ^53^ Our previous findings suggest that the rACC mediates anxiety‐like behaviors associated with both neuropathic pain and inflammatory pain.^25^, ^54^, ^55^ Accordingly, rACC is closely associated with pain and negative emotions.

Therefore, both the BLA and rACC are highly correlated with pain and negative emotions. Our study found that BLA CaMKII neurons project to the rACC. However, it remains uncertain whether the BLA^CaMKII^‐rACC neural circuit plays a role in mediating mechanical allodynia and anxiety‐like behaviors. We first inhibited BLA^CaMKII^‐rACC in sham mice. The results showed mechanical allodynia and anxiety‐like behaviors were induced in sham mice after the BLA^CaMKII^‐rACC was inhibited. To further substantiate the role of the BLA^CaMKII^‐rACC neural circuit, we activated the neural circuit in SNI mice. The results indicated that when the BLA^CaMKII^‐rACC in SNI mice was activated, both mechanical allodynia and anxiety‐like behaviors in SNI mice were alleviated. At the same time, the activity of the BLA CaMKII neurons that projected to the rACC was elevated. The results suggest that BLA^CaMKII^‐rACC mediates the mechanical allodynia and anxiety‐like behaviors in mice.

Our previous findings suggest EA alleviated anxiety‐like behaviors induced by neuropathic pain by regulating BLA.^13^ In addition, EA alleviated anxiety‐like behaviors caused by chronic pain through rACC.^25^ However, it is still unclear whether the analgesic and anxiolytic effects of EA are associated with the BLA^CaMKII^‐rACC neural circuit. Therefore, in the SNI‐mCherry‐CNO‐EA group, we performed EA intervention on the SNI mice. In the SNI‐hM4D‐CNO‐EA group, we first administered CNO to inhibit bilateral BLA^CaMKII^‐rACC in SNI mice, followed by EA intervention.

The results indicated that mechanical allodynia and anxiety‐like behaviors were more severe in the SNI‐hM4D‐CNO‐EA group compared to the SNI‐mCherry‐CNO‐EA group, suggesting that inhibition of the BLA^CaMKII^‐rACC neural circuit antagonized the analgesic and anxiolytic‐like effects of EA. Thus, the analgesic and anxiolytic effects of EA can be inferred to be associated with the BLA^CaMKII^‐rACC neural circuit.

## 5 CONCLUSIONS

In summary, we verified the analgesic and anxiety‐like effects of 2 Hz EA in SNI mice and may be associated with BLA CaMKII neuron activity. Besides, we have demonstrated a neural circuit mechanism in which BLA^CaMKII^‐rACC plays a crucial role in mechanical allodynia and anxiety‐like behaviors. Additionally, BLA^CaMKII^‐rACC may mediate the analgesic and anxiolytic effects of 2 Hz EA.

## AUTHOR CONTRIBUTIONS

Yuerong Chen, Siyuan Tong, Yingling Xu, Yunyun Xu, Zonglin Wu, Xixiao Zhu, Xirui Wang, Chaoran Li, Chalian Lin, Xiaoyu Li, Chi Zhang, Yifang Wang: performed experiments and analyzed data. Yuanyuan Wu, Jianqiao Fang, Xiaomei Shao: design of experimental protocols, supervision and conceptualization. Yuerong Chen, Siyuan Tong: manuscript writing and editing. Yuanyuan Wu, Jianqiao Fang, Yunyun Xu, Yuerong Chen, Xiaoyu Li: funding acquisition.

## FUNDING INFORMATION

This work was funded by the National Natural Science Foundation of China (Grant number: 82074541); Natural Science Foundation of Zhejiang Province (Grant number: LY19H270007, LY23H270009, LQ24H270003); Zhejiang Chinese Medical University (Grant number: 2021YKJ08, 2021YKJ09); and the National College Students’ Innovation and Entrepreneurship Training Program of China (Grant number: 202210344020).

## CONFLICT OF INTEREST STATEMENT

The authors declare that they have no conflict of interest.

## Data Availability

The data that support the findings of this study are available from the corresponding author upon reasonable request.
